# Sertoli cell androgen receptor signalling in adulthood is essential for post‐meiotic germ cell development

**DOI:** 10.1002/mrd.22506

**Published:** 2015-06-09

**Authors:** Ariane Willems, Cornelia Roesl, Rod T. Mitchell, Laura Milne, Nathan Jeffery, Sarah Smith, Guido Verhoeven, Pamela Brown, Lee B. Smith

**Affiliations:** ^1^ Laboratory for Experimental Medicine and EndocrinologyCatholic University of LeuvenLeuvenBelgium; ^2^ MRC Centre for Reproductive HealthUniversity of EdinburghThe Queen's Medical Research InstituteEdinburghUK

Androgens are key drivers of spermatogenesis, and germ cells in mice lacking androgen receptor (AR), specifically from Sertoli cells, arrest in meiosis (reviewed in Smith and Walker, 2014). When Sertoli‐cell AR is ablated during fetal life (De Gendt and Verhoeven, 2012), however, it is impossible to determine whether the meiotic‐arrest phenotype observed in adults results from perturbed Sertoli cell development or perturbed function in adulthood.

We used a lentiviral approach to determine if Sertoli‐cell AR is essential for supporting spermatogenesis specifically in adult testes—an organ where tamoxifen‐inducible knockout may present off‐target effects. Specifically, we introduced Cre recombinase into the Sertoli cells of adult male AR^flox^ mice (De Gendt et al., 2004) to generate adult Sertoli‐Cell AR Knockout (aSCARKO) mice. Lentiviral particles contained both CMV‐Cre recombinase and tRFP635 (red fluorescent protein) transgenes separated by an IRES, or CMV‐tRFP635 alone. Shuttle vectors were packaged using a third‐generation lentiviral vector pseudotyped for VSV‐G, produced at a viral titer of >1 × 10^9^. Virus was introduced into the seminiferous tubules of adult male AR^flox/Y^ via injection into the efferent ducts, using 10 μl of Cre virus, tRFP635 control virus, or optiMEM (vehicle); an additional sham operated, but not injected, control was also evaluted. To control for systemic effects, combinations of Cre/control Cre/optiMEM, Cre/sham, control/optiMEM, control/sham, were generated in testes from individual mice (one treatment per testis; n = 10 per group).

**Figure 1:**
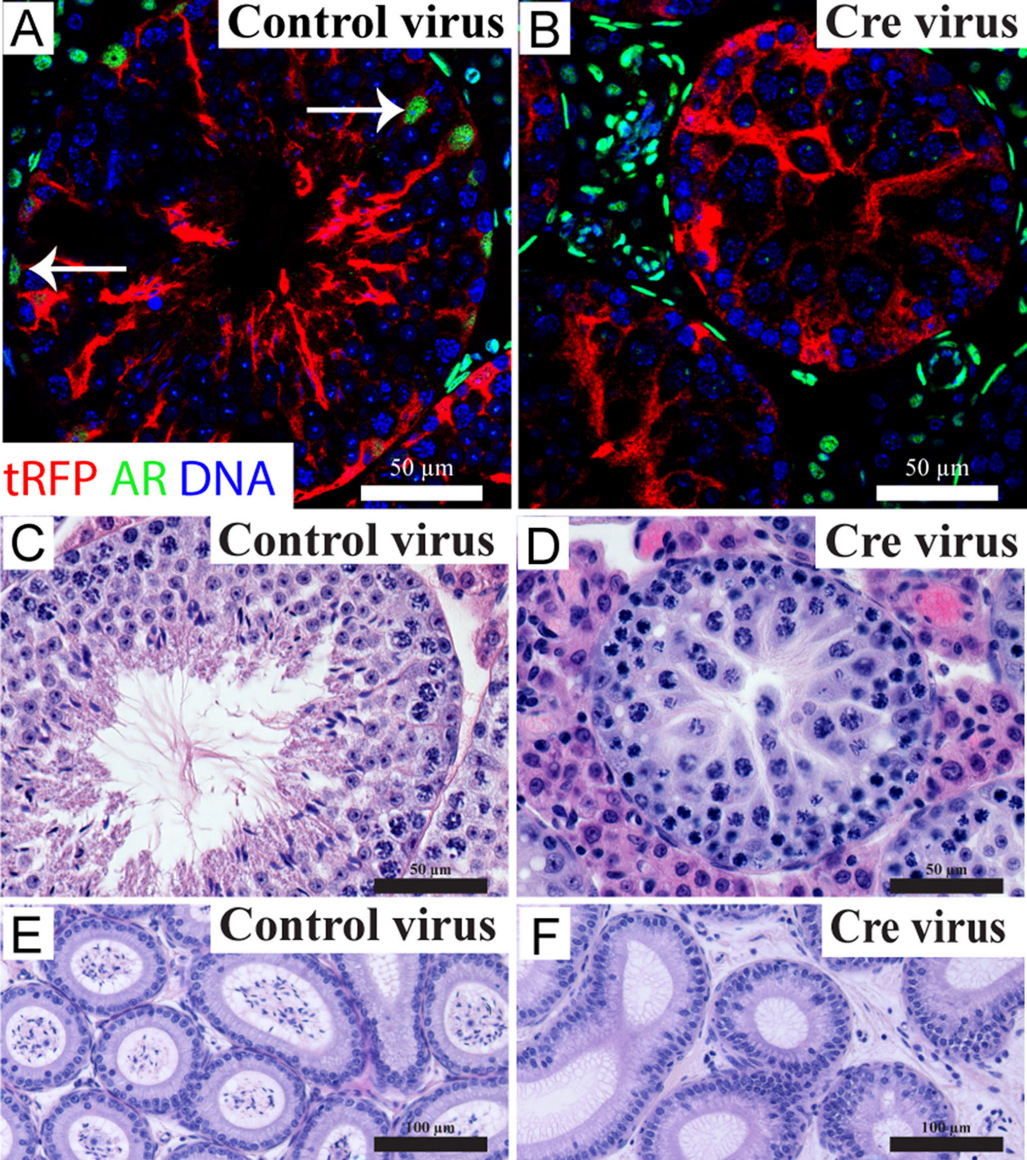
Immunolocalisation of tRFP635 and AR, and testicular and epididymal morphology in virus‐injected adult testes. **A–B:** Sertoli cells retain AR expression (white arrows) following injection of control virus (A), whereas injection of Cre‐encoding virus induces Sertoli cell‐specific ablation of AR (B). **C–F:** Normal testicular (C) and epididymal (E) morphology was observed in controls. Loss of Sertoli‐cell AR following Cre‐encoding virus injection leads to meiotic arrest (D), which resulted in an absence of spermatozoa in the epididymis (F).

Tissues were collected 40 days after surgery (one complete cycle of spermatogenesis). Body and seminal vesicle weight (a biomarker of circulating androgen concentrations) did not differ between any treatment group (data not shown), but the weight of testes injected with Cre recombinase virus was significantly reduced (sham, 92.52 ± 4.29; OptiMEM, 100.12 ± 4.96; tRFP635 control, 70.22 ± 17.31; Cre, 35.48 ± 3.45 mg) to a final weight consistent with developmental‐SCARKO mice (De Gendt et al., 2004). tRFP635 was specifically detected in the cytoplasm of Sertoli cells (Fig. 1A,B), but not in other testicular cell types. AR expression was observed in all somatic cells in both sham‐operated and control tRFP635 lentivirus‐injected testes. In contrast, injection of testes with Cre recombinase virus resulted in Sertoli cell‐specific localisation of tRFP with a loss of AR expression only in Sertoli cells; AR was retained in other testicular somatic cell types. Thus, AR had been selectively ablated in adult Sertoli cells whilst leaving the remainder of the testis untouched.

Forty days post‐injection, seminiferous tubules from control testes retained normal spermatogenesis, with no obvious defects (Fig. 1A,C). Testes injected with Cre recombinase virus, however, displayed evidence of germ‐cell arrest during meiosis (Fig. 1B,D), similar to that observed in SCARKO mice (De Gendt et al., 2004). Furthermore, epididymides continuous with the Cre‐encoding virus‐injected testes contained no mature spermatozoa (Fig. 1F). Therefore, loss of Sertoli‐cell AR in adulthood recapitulates the spermatogenic‐block phenotype observed in developmentally induced SCARKO models, unequivocally demonstrating that Sertoli cell AR is essential for continuous spermatogenesis in adults.
